# Virus-Specific T Cells and Response to Checkpoint Inhibitors in Progressive Multifocal Leukoencephalopathy

**DOI:** 10.1001/jamaneurol.2025.5318

**Published:** 2026-01-20

**Authors:** Nora Möhn, Lea Grote-Levi, Agnes Bonifacius, Sabine Tischer-Zimmermann, Sandra Nay, Konstantin Fritz Jendretzky, Mieke Luise Sassmann, Kevin Karacondi, Melanie Zent, Franz Felix Konen, Kurt-Wolfram Sühs, Sven G. Meuth, Marc Pawlitzki, Clemens Warnke, Ilya Ayzenberg, Ruth Schneider, Christoph Helmchen, Norbert Brüggemann, Stephan Klebe, Marcel Hildner, Christian Grefkes, Louisa Nitsch, Petra Hühnchen, Sebastian Böltz, Laura Alt, Hayrettin Tumani, Christoph Kleinschnitz, Refik Pul, Oliver Grauer, David Clifford, Sharmilee Gnanapavan, Rebecca Wicklein, Thomas Perpoint, Martijn Beudel, Arnaud Del Bello, Sebastian Rauer, Heinz Wiendl, Ilijas Jelcic, Jacques Gasnault, Eleonora Cimini, Andrea Antinori, Carmela Pinnetti, Valérie Pourcher, Nicolas Weiss, Nicolas Lambert, Britta Maecker-Kolhoff, Günter U. Höglinger, Sara Zahraeifard, Irene Cortese, Britta Eiz-Vesper, Guillaume Martin-Blondel, Thomas Skripuletz

**Affiliations:** 1 Department of Neurology, Hannover Medical School, Hannover, Germany; 2 Center of Neurology, Department of Neuroimmunology, University Hospital and University Bonn, Germany; 3 Institute of Transfusion Medicine and Transplant Engineering, Hannover Medical School, Hannover, Germany; 4 Department of Neurology, Medical Faculty and University Hospital Düsseldorf, Heinrich Heine University Düsseldorf, Düsseldorf, Germany; 5 Department of Neurology, Philipps University Marburg, Marburg, Germany; 6 Department of Neurology, University Hospital Gießen and Marburg, Baldingerstraße, Marburg, Germany; 7 Department of Neurology, St Josef Hospital, Ruhr University Bochum, Bochum, Germany; 8 Department of Neurology, University Hospital Schleswig-Holstein, Campus Lübeck, Lübeck, Germany; 9 Department of Neurology, Essen University Hospital, Essen, Germany; 10 Department of Neurology, Goethe University Frankfurt and University Hospital Frankfurt, Frankfurt, Germany; 11 Center of Neurology, Department of Neuroimmunology, University Hospital Bonn, Germany; 12 Department of Neurology with Experimental Neurology, Charité - Universitätsmedizin Berlin, corporate member of Freie Universität Berlin and Humboldt-Universität zu Berlin, Berlin, Germany; 13 Medizinische Klinik 3 - Rheumatologie and Immunologie, Friedrich-Alexander-Universität Erlangen-Nürnberg and Uniklinikum Erlangen, Erlangen, Germany; 14 Deutsches Zentrum für Immuntherapie (DZI), Friedrich-Alexander-Universität Erlangen-Nürnberg and Uniklinikum Erlangen, Erlangen, Germany; 15 Department of Neurology, University Hospital Ulm, Ulm, Germany; 16 Department of Neurology, University Medicine Essen, Essen, Germany; 17 Department of Neurology, University Hospital Münster, Münster, Germany; 18 Department of Neurology, Washington University in St Louis, St Louis, Missouri; 19 Department of Neurology, Barts Health NHS Trust and Queen Mary University of London, London, United Kingdom; 20 Department of Neurology, Technical University of Munich, Munich, Germany; 21 Department of Infectious and Tropical Diseases, Lyon University Hospital, Lyon, France; 22 Amsterdam University Medical Center, University of Amsterdam, Amsterdam, the Netherlands; 23 Department of Nephrology and Organ Transplantation, CHU Rangueil, Toulouse, France; 24 Department of Neurology, Medical Center, University of Freiburg, Freiburg, Germany; 25 Neuroimmunology and Multiple Sclerosis Research Section, Department of Neurology, University Hospital Zurich and University of Zurich, Zurich, Switzerland; 26 Unit of Rehabilitation of Neuroviral Diseases, Bicêtre Hospital, APHP, Le Kremlin-Bicêtre, France; 27 Cellular Immunology and Pharmacology Laboratory, National Institute for Infectious Diseases Lazzaro Spallanzani IRCCS, Rome, Italy; 28 Clinical and Research Department, National Institute for Infectious Diseases Lazzaro Spallanzani IRCCS, Rome, Italy; 29 Department of Infectious Diseases, Pitié-Salpêtrière Hospital, Sorbonne University, Paris, France; 30 Department of Intensive Care Unit, Pitié-Salpêtrière Hospital, Sorbonne University, Paris, France; 31 Department of Neurology, CHU de Liège/Hôpital de la Citadelle, Liège, Belgium; 32 Department of Pediatric Hematology and Oncology, Hannover Medical School, Hannover, Germany; 33 Department of Neurology, LMU University Hospital, Ludwig-Maximilians-Universität (LMU) München, Munich, Germany; 34 German Center for Neurodegenerative Diseases, Munich, Germany; 35 Munich Cluster for Systems Neurology, Munich, Germany; 36 Experimental Immunotherapeutics Unit, National Institute of Neurological Disorders and Stroke, Bethesda, Maryland; 37 Infectious Diseases Department, University Hospital Center of Toulouse, Toulouse, France; 38 Toulouse Institute for Infectious and Inflammatory Diseases, INSERM UMR1291, CNRS UMR5051, University of Toulouse, Toulouse, France; 39 NEO-I3D Research Group, Toulouse University Hospital, Toulouse, France

## Abstract

**Importance:**

Progressive multifocal leukoencephalopathy (PML) is a life-threatening demyelinating disease caused by reactivation of the JC virus (JCV) in immunocompromised patients. While immune checkpoint inhibitors (ICIs) show therapeutic potential, responses vary and predictive biomarkers are lacking.

**Objective:**

To determine whether pretreatment JCV- and/or BK virus–specific T cells in the blood are associated with treatment efficacy.

**Design, Setting, and Participants:**

This retrospective cohort study included 111 patients with PML who were treated with ICIs stratified by peripheral virus-specific T cell presence (ELISpot/flow cytometry) between August 2021 and May 2024, with a median (IQR) follow-up of 7 (1-13) months. Of 112 patients with definite PML across 39 centers, 1 patient refused participation; 111 patients were included.

**Exposure:**

Patients received pembrolizumab (n = 81), nivolumab (n = 28), or atezolizumab (n = 2) per availability and prescribing practices at participating centers.

**Main Outcome and Measures:**

Clinical outcomes, diagnostic parameters, and immune-related adverse events were compared; association of virus-specific T-cell responses with survival was analyzed using the Kaplan-Meier method.

**Results:**

The study cohort consisted of 111 patients (median [IQR] age, 61 [50-70] years; 74 male [66.6%]). Twenty-one patients had detectable virus-specific T cells prior to therapy, 22 were T cell–negative and 68 had an unknown T-cell status. T cell–positive patients showed significantly higher response rates and improved survival compared to both T cell–negative patients (18/21 [86%] vs 5/22 [23%]; *P* < .001; median survival time, none [95% CI, undefined] vs 136.5 days [95% CI, 19 to ∞]; *P* = .002) and those with unknown T-cell status (18/21 [86%] vs 29/68 [43%]; *P* = .001; median survival time, none vs 162 days [95% CI, 66 to ∞]; *P* = .004). They achieved better functional outcomes (median [IQR] modified Rankin Scale score, 3 [2-4] vs 4 [3-6]; *P* = .009) and lower JC viral load in cerebrospinal fluid (median [IQR], 0 copies/mL [0-502.5] vs 2500 copies/mL [0-6900]; *P* = .01) during follow-up compared to T cell–negative patients. Immune-related adverse events were most frequent in T cell–negative patients (10/20 [50%]), including the most severe events, and least frequent in T cell–positive patients (2/20 [10%]) (*P* = .02).

**Conclusions and Relevance:**

Preexisting functional virus-specific T cells were associated with better clinical response, longer survival, and lower toxicity in PML. These findings suggest the likely importance of preexisting antiviral immunity for successful ICI therapy.

## Key Points

**Question:**

Are pretreatment JC virus- and/or BK virus-specific T cells in the blood associated with the efficacy of immune checkpoint inhibitors (ICIs) in progressive multifocal leukoencephalopathy (PML)?

**Findings:**

In this cohort study of 111 patients with PML treated with ICIs, those with detectable virus-specific T cells (n = 21) had significantly higher response rates and longer survival than both T cell–negative patients (n = 22) and those with unknown status (n = 68).

**Meaning:**

These findings suggest that functional virus-specific T cells, as markers of preexisting antiviral immunity, may be associated with better clinical outcomes and reduced toxicity in patients with PML who are treated with ICIs.

## Teaser

This cohort study evaluates the association between pretreatment JC virus– and BK virus–specific T cells and treatment efficacy in progressive multifocal leukoencephalopathy.

## Introduction

Progressive multifocal leukoencephalopathy (PML) is an opportunistic brain infection caused by the JC virus (JCV; also known as human polyomavirus 2, HPyV-2, or JCPyV). Primary infection usually occurs during childhood, and immunoglobulin G antibodies against JCV remain detectable in up to 50% to 65% of adults.^1,2^ PML arises upon viral reactivation in the setting of impaired cellular immunity, such as in HIV or immunosuppressive treatment, via genetically rearranged neurotropic JCV variants that lyse oligodendrocytes in the central nervous system.^3^ Given the lack of effective antivirals, prognosis depends on restoring immune competence. In cases where this is unachievable (eg, lymphoproliferative disorders, primary immunodeficiencies, or organ transplant), mortality is particularly high.^4^ Several strategies to reconstitute cellular immunity have emerged, including transfer of allogeneic virus-specific cells^5^ and immune checkpoint inhibitors (ICIs), such as pembrolizumab, nivolumab, and atezolizumab. This is based on the hypothesis that chronic antigen exposure drives T-cell exhaustion, marked by a loss of polyfunctionality and impaired viral clearance.^6^ Since the first 10 cases published in 2019,^7,8,9^ the use of ICIs in PML has become more widespread.^10,11,12^ Response remains variable, with survival reported in approximately 50% to 60% of treated cases.^5^ By contrast, the 12-month survival rate was 45% in the best supportive treatment cohort of a study^13^ consisting of patients who developed PML at a time when allogeneic virus-specific T-cell therapy or ICI therapy was largely unavailable. In a largest recently published study of total 79 patients with PML who were treated with ICIs—including 38 published and 41 previously unpublished cases—a 1-year survival rate of 51.9% was determined.^14^

Predictive biomarkers of major clinical relevance for identifying ICI-responsive patients remain unavailable. We hypothesize that detectable functional JCV- or cross-reactive BK virus (BKV)–specific T cells, arising from sequence homology with JCV^15^ and both reflecting residual antiviral immunity, may be associated with improved clinical outcomes. This hypothesis, supported by in vitro studies and single case reports,^7,16^ has not yet been evaluated in a multicenter setting. In this retrospective multicenter study, we examined whether pre-ICI JCV- and/or BKV-specific T cells in blood are associated with clinical response, survival, and toxicity in patients with PML.

## Methods

### Study Outline

The study was approved by the local institutional review boards. All patients or representatives gave written consent. The Strengthening the Reporting of Observational Studies in Epidemiology (STROBE) reporting guideline was followed.

We conducted a retrospective survival analysis of 111 of 112 PML patients treated at 39 centers worldwide (eTable 1 in Supplement 1) with pembrolizumab, nivolumab, or atezolizumab targeting PD-1 and PD-L1, respectively. One patient declined to participate. Of the 111 patients included, 21 had detectable virus-specific T cells in the blood before treatment. The survival of these patients was compared with that of 22 patients with confirmed negative virus-specific T-cell status and an additional 68 patients whose virus-specific T-cell status was unknown.

### Patients

All included patients were adults aged 18 years and older. Patients were treated between August 2021 and May 2024 with pembrolizumab (n = 81), nivolumab (n = 28), or atezolizumab (n = 2). All had definite PML per American Academy of Neurology diagnostic criteria.^17^

### Determination of the Presence of Virus-Specific T Cells in the Patients’ Blood

A total of 43 patients underwent assessment for functional (cytokine-producing) endogenous virus-specific T cells in peripheral blood. In 36 cases, the IFN-γ enzyme–linked immunospot (ELISpot [Lophius Biosciences]) assay was performed, while the remaining 7 patients were evaluated using flow cytometry. A positive ELISpot response was defined as 2 or more spots, provided the negative control showed 0 spots. If the negative control had more than 0 spots, a response was considered positive if it exhibited 2 or more spots above twice the number of spots in the negative control.

For flow cytometry, virus-specific CD4^+^/CD8^+^ T cells were IFN-γ^+^/TNF^+^ after JCV peptide stimulation. Background was determined using unstimulated controls, and responses were considered positive when cytokine-producing cells were 0.1% or more above background. The 68 patients with unknown T-cell status were not tested due to logistical constraints, as some centers lacked access to fresh blood samples and the capacity for T-cell analysis before ICI treatment. Further methodological details are provided in the eMethods in Supplement 1.

### Follow-Up Procedures

ICI treatment and clinical work up was part of routine care and performed by treating neurologists or infectiologists at each center. The clinical status was assessed using the modified Rankin Scale (mRS). Changes in mRS scores were tracked longitudinally, from initiation of ICI therapy to the last follow-up visit, alongside monitoring of JC viral load in the cerebrospinal fluid (CSF) and magnet resonance imaging (MRI) lesion burden throughout the observation period. Therapy response was defined as improvement or stabilization of neurological symptoms regarding mRS. ICI-associated adverse events and PML-immune reconstitution inflammatory syndrome (PML-IRIS) were documented.

### Statistical Analysis

Statistical analyses were performed using GraphPad Prism version 10. Continuous variables were expressed as medians with IQRs. Fisher exact or χ^2^ tests were used for categorical variables. The Kruskal-Wallis test was used for continuous nonnormal variables (eg, JC viral load in CSF), with Dunn multiple comparisons test including Bonferroni correction used for post hoc analysis.

The Mann-Whitney *U* test was used for pairwise group comparisons and included Bonferroni correction. Univariate logistic regression was applied for predictor analysis and the Cox regression method for association between JC viral load and survival. Survival analysis was conducted using the Kaplan-Meier method, and differences between groups were assessed with the log-rank test. Significance was set at *P* < .05, consistent with the assumptions made in the other analyses.

## Results

### Patient Characteristics

The study comprised 111 patients with PML receiving ICI (median [IQR] age, 61 [50-70] years; 74 male [66.6%] and 37 [33.4%] female). Of these patients, 21 (18.9%) had detectable virus-specific T cells prior to the ICI initiation, 22 (19.8%) had no detectable T-cell response, and 68 (61.3%) had an undetermined T-cell status. ElISpot data are shown in eFigure 1 in Supplement 1 as representative images and summarized as an overview of positive findings in eTable 2 in Supplement 1. The median follow-up period for patients with detectable virus-specific T cells in the blood was 12 months (IQR, 6-15; range, 1-51). The median follow-up period was 3.5 months (IQR, 1.75-13.75; range, 0-49) for the group without virus-specific T-cell detection and 8 months (IQR, 1-13.25; range, 1-66) for the group with unknown T-cell status.

Baseline characteristics (Table 1) were comparable across the 3 groups. There were no significant differences in terms of age, sex distribution, underlying diseases, previous immunosuppressive therapy, or the method of PML diagnosis (CSF analysis or brain biopsy). Among patients with a negative JCV polymerase chain reaction result (3 patients with positive T-cell status and with unknown T-cell status), definite PML was confirmed by brain biopsy.^17^

**Table 1.** Baseline Characteristics and Clinical Parameters of Patients With Progressive Multifocal Leukoencephalopathy (PML) Treated With Immune Checkpoint Inhibitors (ICIs), Stratified by JC Virus (JCV)– and BK Virus (BKV)–Specific T-Cell Status

Characteristic	JCV/BKV-specific T cells, No. (%)			*P* value^a^
	Positive (n = 21)	Negative (n = 22)	Status unknown (n = 68)	
Age at PML diagnosis, median (IQR), y	61 (46-68)	59 (45-63)	65 (52-73)	.11
Sex				
Male	15 (71)	14 (64)	45 (66)	.92
Female	6 (29)	8 (36)	23 (34)	
Underlying disease				
Lymphoproliferative	9 (42)	10 (45)	30 (44)	>.99
Autoimmune	0	2 (9)	5 (7)	.56
AIDS	6 (29)	4 (18)	7 (10)	.11
Multiple sclerosis	0	0	2 (3)	>.99
Other lymphopenia	2 (9.5)	3 (14)	12 (18)	.81
Other^b^	2 (9.5)	1 (4.5)	6 (9)	.89
Combination^c^	2 (9.5)	2 (9)	6 (9)	>.99
Previous immunosuppressive therapy				
Yes	8 (38)	14 (63)	43 (63)	.11
No	13 (62)	8 (37)	25 (37)	
Diagnosis confirmed by brain biopsy				
Yes	5 (24)	3 (14)	20 (29)	.36
No	16 (76)	19 (86)	48 (71)	
mRS score at start of ICI treatment, median (IQR)	3 (3-4)	3 (2-4)	3 (2-4)	.47
No. of brain lobes affected (per MRI) before start of ICIs, median (IQR)	3 (2-5)	3 (2-4)	3 (2-4)	.89
JC viral load in CSF at diagnosis/at start of ICIs, median (IQR), copies/mL	703 (199-5012)	15 849 (3450-50 118)	897 (238-22 870)	.002^d^
Time between PML diagnosis and start of ICIs, median (IQR), d	51 (34-74)	21 (13-35)	19.5 (7-41)	.001^d^
No. of ICI cycles, median (IQR)	3 (2-3)	2 (1-3)	3 (2-4)	.19
ICI used				
Pembrolizumab	21 (100)	19 (86)	41 (60)	<.001^d^
Nivolumab	0	3 (14)	25 (37)	<.000^d^
Atezolizumab	0	0	2 (3)	>.99

Abbreviations: CSF, cerebrospinal fluid; MRI, magnetic resonance imaging; mRS, modified Rankin Scale.

^a^ *P* values indicate statistical comparisons between patients.

^b^ Other included: for virus-specific T-cell–positive patients, 1 patient each with immunoglobulin G deficiency syndrome and unknown status; for virus-specific T-cell–negative patients, 1 patient with cholangiocarcinoma; for virus-specific T-cell status unknown, 1 patient each with hepatocellular carcinoma, prostate cancer, and lung adenocarcinoma and 3 patients with kidney transplant.

^c^ Combination included: for virus-specific T-cell–positive patients, 1 patient each with solid cancer and additional autoimmune or lymphoproliferative disease; for virus-specific T-cell–negative patients, 2 patients with common variable immunodeficiency (other lymphopenia) and additional autoimmune disease; for virus-specific T-cell status unknown, 1 patient each with lymphoproliferative disease and additional common variable immunodeficiency (other lymphopenia) or AIDS and 4 patients with additional solid cancer disease.

^d^ Significant comparison.

A total of 65 patients (65/111 [58.5%]) had received immunosuppressive therapy prior to the diagnosis of PML. For 58 of these individuals, data regarding the continuation or discontinuation of immunosuppression following diagnosis were available. Overall, immunosuppressive treatment was discontinued in 48 patients (83%). Of 17 patients with HIV/AIDS, 7 (41%) developed PML while receiving stable antiretroviral therapy with effective plasmatic viral suppression, and another 9 (53%) had either recently started or were optimizing antiretroviral therapy at diagnosis of PML. In 1 patient, no detailed information was available regarding the status of antiretroviral therapy at the time of PML diagnosis. Following the diagnosis of PML, all patients received optimized antiretroviral therapy, leading to virological control.

### Disease Activity Prior to Therapy

The clinical status, as assessed using the mRS, and the number of brain lobes affected by PML, were comparable across the 3 groups (Table 1). At the start of ICI therapy, patients exhibited moderate disability, with a median (IQR) mRS score of 3 (3-4) in those with detectable virus-specific T cells, 3 (2-4) in those without, and 3 (2-4) in the group with unknown T-cell status. MRI showed a similar extent of brain involvement, with a median (IQR) of 3 (2-5) affected lobes in patients with virus-specific T cells, 3 (2-4) in those without, and 3 (2-4) in the group with unknown status. The JC viral load in CSF differed between the groups (Table 1). Patients without virus-specific T cells had a significantly higher viral load (median [IQR], 15 849 [3450-50 118] copies/mL) compared to both patients with virus-specific T cells (703 [199-5012] copies/mL; *P* = .002) and those with an unknown T-cell status (897 [238-22 870] copies/mL; *P* = .02). No significant difference was observed between patients with virus-specific T cells and those with unknown status.

### Therapy Characteristics

Table 1 details the therapeutic characteristics of the 111 patients treated. Patients with detectable virus-specific T cells started ICI treatment later following diagnosis, with a median (IQR) of 51 (34-74) days compared to 21 (13-35) days (*P* = .008) in those without detectable virus-specific T cells and 19.5 (7-41) days (*P* = .001) in those with unknown T-cell status.

The number of infusion cycles was similar, but the allocation of ICI therapy differed significantly between them. In patients with virus-specific T cells, pembrolizumab was the sole ICI administered, while it was used in 19 of 22 patients (86%) without virus-specific T cells and in 41 of 68 (60%) of those with unknown T-cell status (*P* < .001). Conversely, nivolumab was more frequently used in patients without virus-specific T cells (3/22 [14%]) and in 25 of 68 (37%) of those with unknown status, while it was not used in any patient with virus-specific T cells (*P* < .001). The differences regarding the specific ICI product are most likely due to their availability or prescription practices in the respective centers.

### Response to Therapy

**Figure 1.**
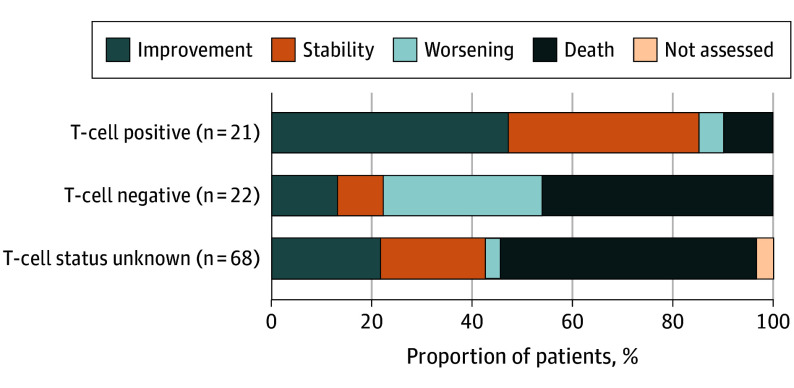
Proportion of Patients With Clinical Improvement, Stability, Worsening, or Death, Stratified by Presence of JC Virus– and BK Virus–Specific T-Cell Responses Before Therapy

Patients, who died from PML or exhibited continuous deterioration without a phase of clinical stabilization or without signs of inflammatory PML on MRI (potential PML-IRIS) were classified as nonresponders. Among 21 patients with detectable virus-specific T cells, 18 (86%) responded to ICI therapy (Figure 1; Table 2). One patient experienced a worsening of neurological symptoms, which was most likely due to PML-IRIS. Two others died from their underlying disease 35 and 201 days after starting ICI.

**Table 2.** Follow-Up Characteristics and Clinical Parameters of Patients With Progressive Multifocal Leukoencephalopathy (PML) Treated With Immune Checkpoint Inhibitors (ICIs), Stratified by JC Virus (JCV)– and BK Virus (BKV)–Specific T-Cell Status

Characteristic	JCV/BKV-specific T cells			*P* value^a^
	Positive (n = 21)	Negative (n = 22)	Status unknown (n = 68)	
Outcome at last follow-up				
Death due to PML	0	10 (45)	32 (47)	<.001^b^
Death due to underlying disease	2 (10)	0	3 (4)	.29
Alive, PML symptoms stable	8 (38)	2 (9)	14 (21)	.08
Alive, PML symptoms improved	10 (48)	3 (14)	15 (22)	.04^c^
Alive, PML symptoms worsened	1 (4)	7 (33)	2 (3)	.001^b^
NA^d^	0	0	2 (3)	NA
JC viral load in CSF at last follow-up, median (IQR), copies/mL	0 (0-154)	1100 (0-40 000)	609 (0-59 852)	.01^b^
mRS score at last follow-up, median (IQR)	3 (2-4)	4 (3-6)	NA^e^	.009^b^
No. of brain lobes affected (per MRI) at last follow-up, median (IQR)	3 (2-4)	3 (3-5)	4 (2-6)	.44
PML-IRIS				
Yes	4 (19)	0	14 (21)	.07
No	16 (76)	20 (91)	54 (79)	
NA^b^	1 (5)	2 (9)	0	
Adverse effects of ICI				
Yes	2 (10)	10 (45.5)	20 (29)	.02^b^
No	18 (85)	10 (45.5)	48 (71)	
NA^d^	1 (5)	2 (9)	0	

Abbreviations: CSF, cerebrospinal fluid; IRIS, immune reconstitution inflammatory syndrome; MRI, magnetic resonance imaging; mRS, modified Rankin Scale; NA, not applicable.

^a^ *P* values indicate statistical comparisons between patients.

^b^ Significant comparison.

^d^ For IRIS and adverse event reporting, NA in the T cell–positive (1/21) and T cell–negative (2/22) groups indicates patients without follow-up information on these parameters. Outcome at last follow-up was not documented in 2 patients of the unknown T-cell group.

^e^ Data not available due to incomplete documentation at some centers. Specifically, mRS score at last follow-up was missing in parts of the unknown T-cell status group (mRS data available for 31/68 patients).

In the virus-specific T cell–negative group, 10 of 22 patients (45%) did not respond and died from PML within a median (IQR) of 55 (15-104) days after the start of ICI treatment. Among the 12 survivors, 7 (58%) experienced a deterioration in neurological symptoms without signs of inflammatory PML on MRI, classifying them as additional nonresponders. Ultimately, 5 of 22 patients (23%) in the virus-specific T cell–negative group responded to ICI treatment.

In the group of patients with unknown T-cell status, 29 of 68 (43%) responded to therapy, showing either improvement or stable symptoms (Figure 1). Eight nonresponders within the group of unknown T cell-status had a documented PML-IRIS and died during the course of PML disease, but the exact relationship to death remained unclear. No PML-IRIS–associated deaths occurred within the group of virus-specific T cell–positive or –negative nonresponders.

**Figure 2.**
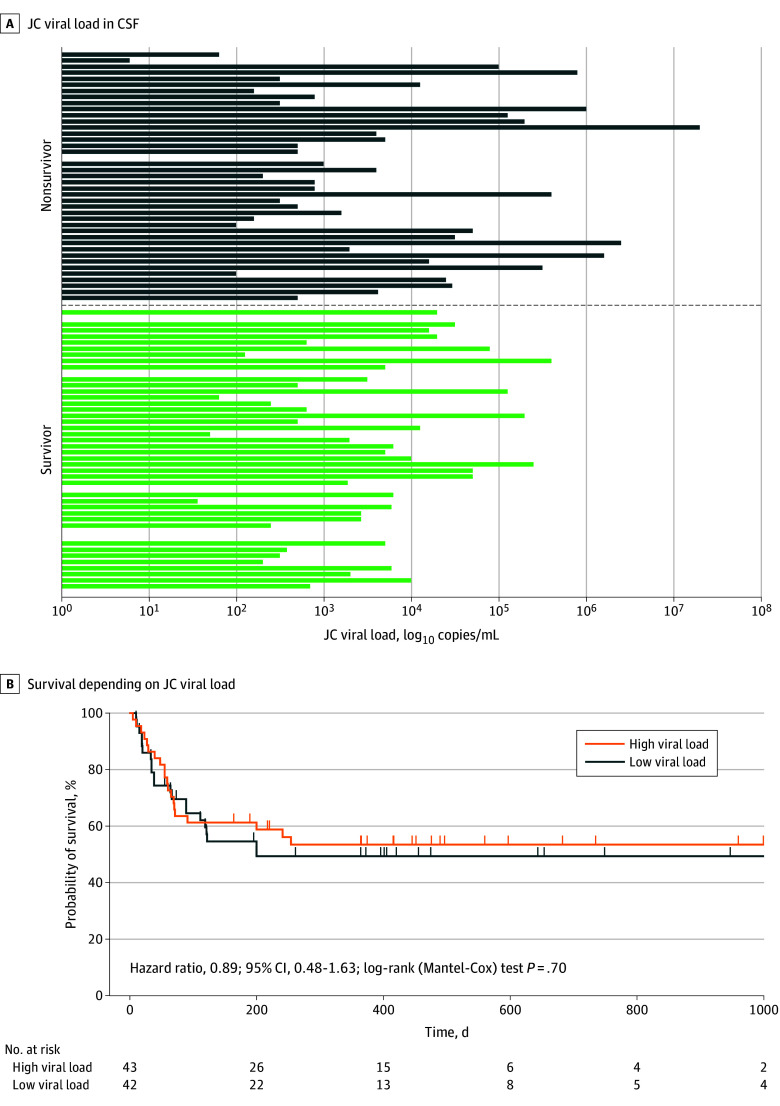
Comparison of Survivor and Nonsurvivor Depending on JC Virus Load Prior to Immune Checkpoint Inhibitor (ICI) Treatment Initiation A, Comparison of JC viral load in cerebrospinal fluid between survivor and nonsurvivor: of 111 patients treated with immune checkpoint inhibitors (ICIs), data of absolute JC viral load in cerebrospinal fluid prior to therapy initiation of survivors (n = 46) and nonsurvivors (n = 41) are presented. B, Survival depending on JC viral load: the red curve (n = 44) represents patients with a high JC viral load in cerebrospinal fluid (above the median of >2531 copies/mL) prior to initiation of ICI therapy; the blue curve (n = 43) represents those with a low viral load. During the disease course, 20 patients with high JC viral load and 21 with low JC viral load died. No significant difference in overall survival was observed between the 2 groups.

With regard to the variability of viral load before therapy initiation, no significant association was found between JC viral load prior to therapy and survival-time in patients who died after ICI initiation (n = 30; ρ, −0.13; 95% CI, −0.43 to 0.19; *P* = .41). The comparison of survivors (n = 46) and nonsurvivors (n = 41) based on JC viral load in CSF prior ICI treatment (Figure 2A) showed no significant difference (median [IQR], 2695 [251-13 404] copies/mL vs 1995 [316-75 059] copies/mL; *P* = .39). Furthermore, no significant difference in overall survival was found for patients with high vs low viral load prior to therapy using Kaplan-Meier survival analysis of the total cohort (median split of JC viral load, 2531 copies/mL; *P* = .70) (Figure 2B).

In the total cohort, only pretreatment CSF JC viral load showed a statistically significant but clinically negligible association with therapeutic response (odds ratio [OR], 0.999997; 95% CI, 0.999991-0.999999; *P* = .007). No other predictors showed significance (eTable 3 in Supplement 1). Within the unknown T-cell status subgroup, higher baseline disability, as measured via mRS, was the only predictor associated with reduced odds of treatment response (OR per 1-point increase, 0.59; 95% CI, 0.35-0.95; *P* = .03) (eTable 4 in Supplement 1).

Following ICI-therapy, the CSF JC viral load was significantly lower in patients with detectable virus-specific T cells (median [IQR], 0 [0-154] copies/mL) compared to those without virus-specific T cells (median [IQR], 1100 [0-40 000] copies/mL; *P* = .03), as well as in those with an unknown T-cell status (median [IQR], 609 [0-59 852] copies/mL; *P* = .01). Functional outcomes at follow-up significantly differed as well. Patients with virus-specific T cells exhibited a stable mRS score (median [IQR], 3 [2-4]), whereas those without specific T cells experienced worsening disability (median [IQR], 4 [3-6]; *P* = .05). MRI findings with respect to affected lobes did not exhibit statistically significant changes across the groups during follow-up (Table 2).

### Survival Analysis

**Figure 3.**
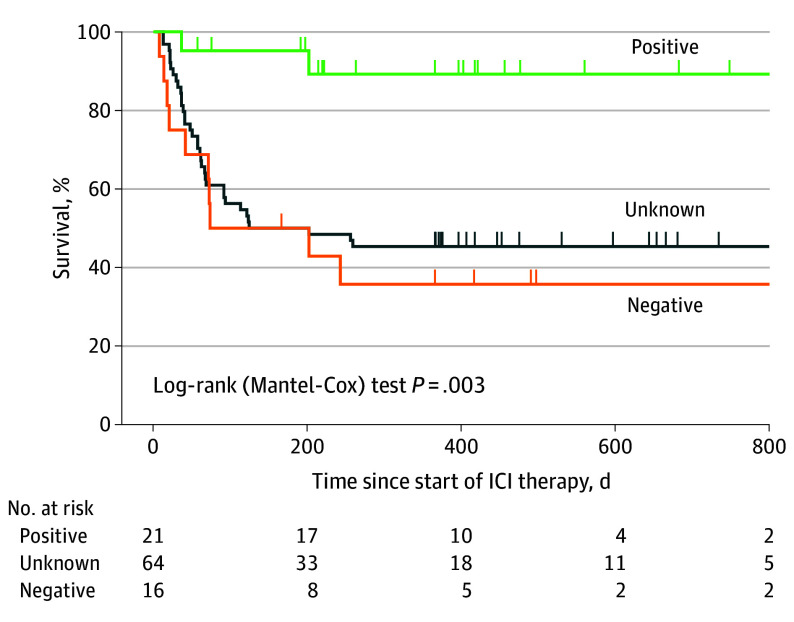
Kaplan-Meier Survival Curves for Patients With Progressive Multifocal Leukoencephalopathy Treated With Immune Checkpoint Inhibitors (ICIs) According to Virus-Specific T-Cell Status The blue curve (n = 21) represents patients with detectable virus-specific T cells before ICI therapy, the gray curve (n = 64) represents patients with unknown T-cell status, and the red curve (n = 16) represents patients without detectable virus-specific T cells prior to ICI therapy initiation. In total, 8 patients who deteriorated despite ICI treatment (2 with unknown T-cell status and 6 from the T-cell–negative group) subsequently received an experimental approach using allogeneic virus-specific T cells following ICI therapy. Since this constituted a distinct therapeutic strategy, these patients were excluded from the survival analysis. For 2 patients with unknown T-cell status, survival was not assessed.

Kaplan-Meier survival analysis (Figure 3) demonstrated a significant survival advantage in patients with detectable virus-specific T cells prior to ICI therapy compared to those without virus-specific T cells (hazard ratio [HR], 0.11; 95% CI, 0.04-0.37; log-rank test *P* = .002), and with unknown virus-specific T-cell status (HR, 0.14; 95% CI, 0.07-0.28; log-rank test *P* = .004). The 1-year survival probability was highest in T cell–positive patients (89.3%), whereas the negative (35.7%) and unknown-status (45.3%) groups exhibited significantly lower survival rates.

Median survival was not reached during the observation period in patients with detectable virus-specific T cells, indicating a potential survival advantage in this group. In contrast, the median [95% CI] survival time for T cell–negative patients was 136.5 (19-∞) days, while the unknown-status group had a median (95% CI) survival time of 162 (66-∞) days. No significant difference was observed between the virus-specific T cell–negative and unknown-status groups (HR, 1.26; 95% CI, 0.60-2.68; log-rank test *P* = .51).

### Tolerability of Immune Checkpoint Inhibitors

PML-IRIS occurred in 4 of 21 patients (19%) with detectable virus-specific T cells, in 0 of 22 of those without virus-specific T cells, and in 14 of 68 (21%) of those with an unknown T-cell status, exhibiting a trend but no statistical significance (*P* = .07).

Adverse effects related to ICI therapy were most frequent in patients without virus-specific T cells (10/20 [50%]), followed by those with an unknown T-cell status (20/68 [29%]), and least frequent in patients with virus-specific T cells (2/20 [10%]) (*P* = .02). In the group with unknown T-cell status, 3 patients experienced more than 1 adverse event. Of the 32 patients who experienced adverse events, 20 (62.5%) had received prior immunosuppressive therapy. In the overall cohort, the proportion of patients with prior immunosuppression was 60% (65/111). Thus, no relevant difference in adverse events was observed regarding prior immunosuppressive therapy. Detailed information on treatment-related adverse effects is provided in eTable 5 in Supplement 1.

## Discussion

This cohort study demonstrates that the presence of JCV- or BKV-specific T cells in the blood of patients with PML was associated with improved outcomes after ICI therapy. To evaluate treatment response, we included clinical and laboratory parameters such as functional outcome (mRS score) and CSF JC viral load, in addition to survival, since survival alone does not fully reflect therapeutic success. Patients who had detectable virus-specific T cells before treatment showed higher response rates, better functional recovery, lower CSF JC viral loads during follow-up, and longer survival than those without a pretreatment detectable virus-specific T cell response. These findings highlight the potential importance of residual cellular immunity for successful ICI therapy in PML.

The presence of virus-specific T cells likely reflects preserved endogenous antiviral immunity, which itself contributes to better clinical outcomes. This may introduce a potential confounding effect, as patients with stronger baseline immune competence could have a survival advantage independent of ICI treatment. Nevertheless, ICIs were administered only to patients with progressive PML despite the presence of residual virus-specific T-cell function. The more favorable outcomes observed in these patients are therefore likely related to the presence of exhausted but functionally restorable T cells that can be reactivated by ICI therapy. Remodeling of T-cell exhaustion represents a central mechanism targeted by ICI, and our data suggest that such reactivation is possible in PML.

ICIs, such as nivolumab and pembrolizumab, are primarily used in oncology to enhance CD8+ T cell activity and strengthen the antitumor response, but their mechanisms are also relevant in chronic viral infections.^18,19^ The PD-1/PD-L1 pathway, which is initially upregulated during acute infection to limit tissue damage, can promote T-cell exhaustion in persistent infection, as shown in HIV, hepatitis B, and hepatitis C.^20,21,22^ This process leads to a gradual loss of effector function, diminished cytokine production, and impaired viral control.^23^ In PML, this concept gained particular attention because elevated PD-1 expression has been observed in T cells in both blood and CSF.^7,24^ Moreover, increased PD-1/PD-L1 expression has been demonstrated in autopsy PML lesions.^7^ These findings provided the rationale for ICI use in PML to reverse T-cell exhaustion and restore virus-specific immune responses.^25^

However, our data suggest that ICI therapy might be less effective and may even be harmful in the absence of virus-specific T cells. This observation aligns with findings in oncology and chronic infections, where a preexisting T-cell response appears essential for ICI efficacy.^26^ A previous PML study^27^ likewise associated JCV-specific cytotoxic T cells with early viral control.

ICI-related toxicities varied across the cohort. Patients with detectable virus-specific T cells showed the lowest rates of immune related adverse events, whereas T cell–negative patients had the highest, including severe cases, such as autoimmune myositis, hemolytic anemia, and cardiac toxicity. This pattern suggests that preserved antiviral immunity may mitigate the risk of severe immune toxicity, possibly through more regulated immune reconstitution. Mechanistically, PD-1 blockade may reactivate functionally exhausted cells, promoting a targeted antiviral response rather than broad immune activation.^28^ In contrast, in patients lacking virus-specific T cells, the absence of antigen specific control may lead to nonspecific immune activation, resulting in off target toxicity and autoimmunity.^29^

Aside from T-cell presence and viral load, no other clinical predictors of treatment response were identified. Baseline disability (mRS) was comparable between groups. However, patients with detectable virus-specific T cells had lower baseline CSF JC viral loads before ICI therapy than those without detectable T cells or with unknown T-cell status. Although this finding may indicate partial viral control through residual immunity, baseline viral load neither was neither associated with survival nor predicted meaningful clinical outcomes.

The results of this study support pretreatment assessment of virus-specific T cells as a promising biomarker for identifying patients with PML most likely to benefit from ICI therapy. Stratification time did not influence treatment response among T cell–positive patients. Assays such as ELISpot or flow cytometry could be implemented analogously to PD-L1 testing in oncology to identify candidates for ICI therapy.^30^ For patients without detectable virus-specific T cells, alternative strategies, such as adoptive transfer of donor-derived JCV- or BKV-specific T cells, may be considered when available.^13^

### Limitations

This study has several limitations. Given its retrospective design, evaluations were not standardized across centers, and virus-specific T-cell testing was available only for a subset of patients. Due to logistical constraints of T-cell analysis and given the rapid progression of PML, several centers opted to start checkpoint inhibitor therapy without pretreatment T-cell testing.

Although knowing the T-cell status for all patients would allow a direct comparison of both groups, our results show the probability of response to checkpoint inhibitor therapy even when this status is unknown. This makes this patient group valuable for drawing conclusions about treatment efficacy. Some follow-up and adverse event data were incomplete. Nevertheless, the multicenter design and consistent direction of results across sites strengthen the validity of the findings.

## Conclusions

In conclusion, this study identifies strategies to improve patient selection for ICI therapy in PML and underscores the importance of preexisting antiviral immunity that can be reactivated by treatment. The presence of virus-specific T cells was associated with improved survival, better functional recovery, and lower CSF JC viral loads. However, the absence of detectable virus-specific T cells or limited assay availability should not preclude or delay ICI therapy when clinically justified in the context of a potentially life-threatening disease. Clinical benefit observed in some patients despite undetectable T-cell responses suggests that ICI therapy may exert additional effects beyond the reactivation of preexisting immunity. Alternatively, these findings may reflect limitations in the sensitivity of current detection methods, with some patients retaining residual T-cell activity below the detection threshold.

## Notes

Supplement 1. eMethods eFigure 1. Representative ELISpot data of two patients eFigure 2. ICS gating strategy and representative data of one patient eTable 1. Overview of treatment centers eTable 2. Overview of positive ELISpot findings eTable 3. Outcome predictors of therapeutic response in the overall cohort eTable 4. Outcome predictors of therapeutic response within the unknown T-cell cohort eTable 5. Adverse events eReferences

Supplement 2. Immunotherapy for PML Study Group members

Supplement 3. Data sharing statement
