# Supplementary material for: Virus-Specific T Cells and Response to Checkpoint Inhibitors in Progressive Multifocal Leukoencephalopathy
Source: JAMA Neurol. 2026 Jan 20;83(3):280–9. doi: 10.1001/jamaneurol.2025.5318 (PMC12820779; doi:10.1001/jamaneurol.2025.5318)
Supplement: Supplement 1. — eMethods eFigure 1. Representative ELISpot data of two patients eFigure 2. ICS gating strategy and representative data of one patient eTable 1. Overview of treatment centers eTable 2. Overview of positive ELISpot findings eTable 3. Outcome predictors of therapeutic response in the overall cohort eTable 4. Outcome predictors of therapeutic response within the unknown T-cell cohort eTable 5. Adverse events eReferences [file jamaneurol-e255318-s001.pdf]

Supplemental Online Content

Möhn N, Grote-Levi L, Bonifiacus A, et al; Immunotherapy for PML Study Group. Virus-specific t cells and response to checkpoint inhibitors in progressive multifocal leukoencephalopathy. *JAMA Neurol*. Published online January 20, 2026. doi:10.1001/jamaneurol.2025.5318

eMethods
eFigure 1. Representative ELISpot data of two patients
eFigure 2. ICS gating strategy and representative data of one patient
eTable 1. Overview of treatment centers
eTable 2. Overview of positive ELISpot findings
eTable 3. Outcome predictors of therapeutic response in the overall cohort
eTable 4. Outcome predictors of therapeutic response within the unknown T-cell cohort
eTable 5. Adverse events
eReferences

This supplemental material has been provided by the authors to give readers additional information about their work.

© 2026 Möhn N. et al. *JAMA Neurology*.

eMETHODS

ELISPOT

Presence and frequency of virus-specific T cells in the peripheral blood of patients were determined by ELISpot assay as described before.[1] In brief, after overnight resting in RPMI Medium (Lonza) supplemented with 10% human AB serum (c.c.pro), isolated Peripheral Blood Mononuclear Cells (PBMCs) were stimulated with overlapping peptide pools covering the whole sequence of the viral protein 1 (VP1) and the large T (LT) protein (PepTivator BKV_VP1, BKV_LT, JCV_VP1 and JCV_LT; Miltenyi Biotec) at a final concentration of 1 µg/mL of each peptide overnight. Unstimulated PBMCs served as negative control, while PBMCs stimulated with 1 µg/mL staphylococcal enterotoxin B (SEB, Millipore Sigma) or a mitogen, PHA 10 µg/mL (Sigma) served as positive control. IFN-γ secretion was detected using an AID iSpot Reader System and AID ELISpot software version 8.0 (Autoimmun Diagnostika). In Italy, Spots were counted, expressed and normalized as the number of spots forming cells (SFC)/10^6 PBMC. In Germany, spots were counted and expressed as the number of spots per well (spw)/5.0×10^5 PBMCs. Values obtained from negative control were subtracted.

FLOW-CYTOMETRY

Virus-reactive T cells were expanded using commercially available overlapping peptide libraries against immunodominant viral antigens obtained from commercial vendors (JPT and Miltenyi). Cryopreserved PBMC were thawed and pulsed with peptide libraries (final concentration of 1µg/ml). Cells were suspended in AIM-V media (Thermo-Fisher) supplemented with inactivated 5% human serum and IL-7 (10ng/ml, Peprotech), and plated on 96 well U-bottom plates. Cultures were maintained, fed, and split as needed, every 3 days for approximately 14 days, with IL-2 (30 IU/ml, Peprotech) added 72 hours after the initial stimulation. Cultures were re-stimulated with the same peptide libraries for 4-6 hours in presence of Brefeldin A and Monensin A (BD Biosciences) per manufacturer's instructions. Intracellular cytokine production was measured by flow cytometry. Cells were prepared using BD Cytofix/Cytoperm Kit following manufacturer's instructions. The following surface markers and cytokines were analyzed: viability (Vivid, Invitrogen/Molecular Probes), CD4, CD8, TNFα, IFN-ϒ, IL-2 and granzyme B.

DEFINITION OF T CELL POSITIVITY IN BLOOD

For ELISpot, a response was considered positive when the number of spots was ≥2 above background (unstimulated wells), according to pre-established in-house protocols for detecting low-frequency responses. In cases where the negative control (NC) was >0, a response was considered positive if it exceeded 2 spots above 2×NC.[2,3,4]

For flow cytometry, virus-specific CD8+ and/or CD4+ T cells were defined as cytokine-producing (IFN-γ+/TNF+) cells upon stimulation with overlapping JCV peptide pools. Unstimulated controls were performed to determine background reactivity. A positive result was obtained when the specific cytokine-positive cells reached a frequency of 0.1% above background within the CD4/CD8-positive T cells.

Gating strategy and a representative example of ICS data are shown in eFigure 2.

The 68 patients with an "unknown" status were not tested, as such analyses were not currently standard of care, and retrospective evaluation was not feasible due to the lack of available material.

eFigure 1: Representative ELISpot data of two patients

© 2026 Möhn N. et al. *JAMA Neurology*.

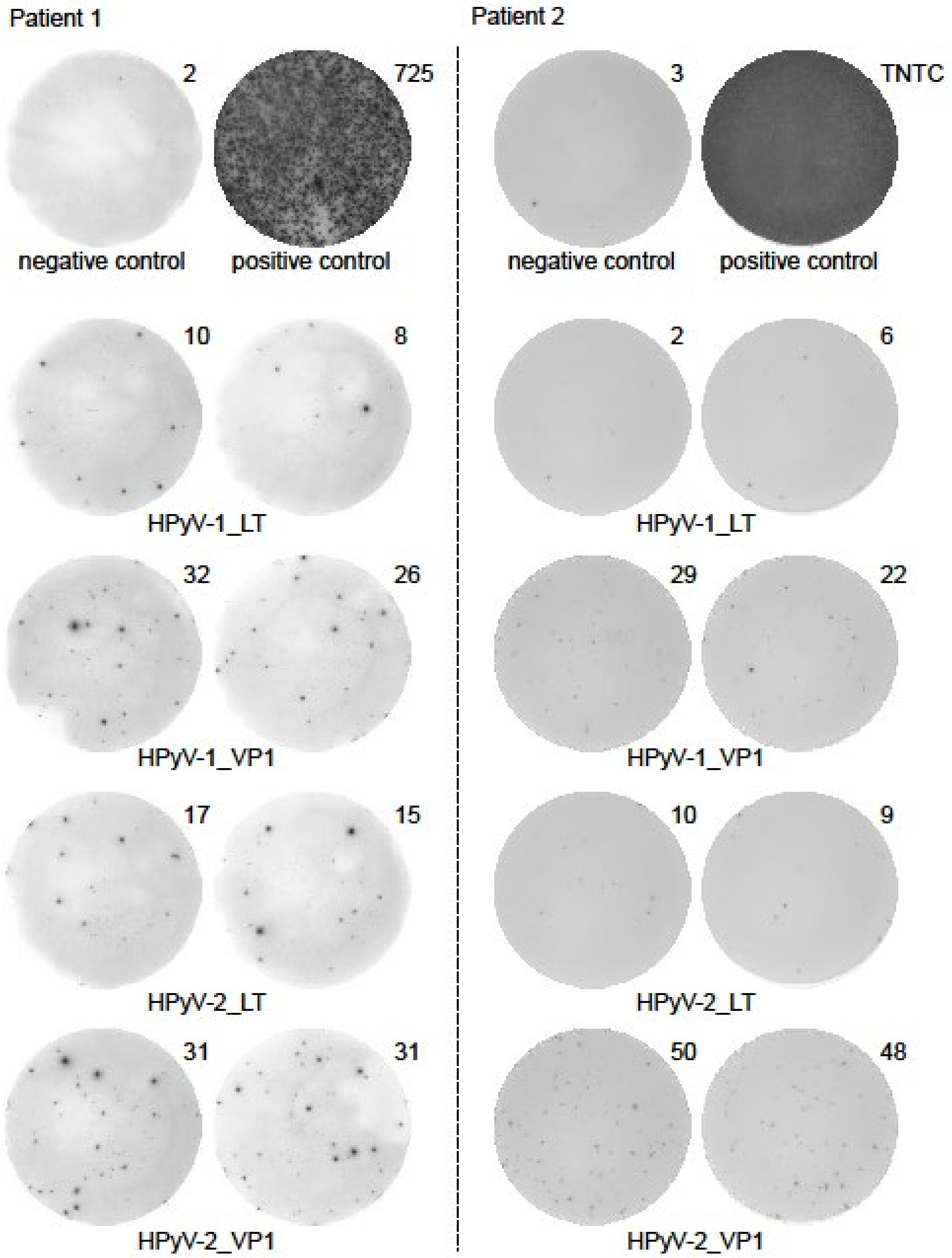

Representative EliSpot data of two patients. Each condition is shown as technical duplicates (two wells per stimulus). Negative and positive controls are shown as single wells. Antigen-specific stimulation was performed using BKV_LT, BKV_VP1, JCV_LT and JCV_VP1 peptides. Number explaining spots per well. Abbreviations: TNT=too numerous to count. JCV: JC virus; BKV: BK virus; VP1: viral protein 1; LT= large T protein.

eFigure 2: ICS gating strategy and representative data of one patient
A

© 2026 Möhn N. et al. *JAMA Neurology*.

B

ICS gating strategy and representative data of one patient.

A. Gating was performed sequentially on lymphocytes à singlets à viable CD45+ cellsà CD3+ cells à

CD4+/CD8 subsets, as shown for unstimulated (Neg) and viral peptide-stimulated cells (T-Ag and VP1 peptide pools, respectively).

B. Representative ICS data showing IFNg/TNF production in unstimulated (Neg) and viral peptide-stimulated cells (T-Ag and VP1 peptide pools, respectively).

eTable 1: Overview of treatment centers

© 2026 Möhn N. et al. *JAMA Neurology*.

Center	total patients treated/center	JCV/BKV-specific T cells positive (n=21)	JCV/BKV-specific T cells negative (n=22)	JCV/BKV-specific T cell status unknown (n=68)
Hannover, Germany	16	10	6	
Paris, France	15		3	12
Bethesda, United States of America	11	4	2	5
Rome, Italy	6	1	4	1
Bonn, Germany	5		1	4
Toulouse, France	5			5
Freiburg, Germany	5			5
Bordeaux, France	3			3
Munich, Germany	3			3
Zurich, Switzerland	3			3
Amsterdam, the Netherlands	2			2
Berlin, Germany	2		2	
Bochum, Germany	2	2		
Cologne, Germany	2			2
Erlangen, Germany	2	1	1	
Liège, Belgium	2			2
London, United Kingdom	2			2
Lyon, France	2			2
Münster, Germany	2		1	1
Saint Louis, United States of America	2			2
Aurora, United States of America	1			1
Bayonne, France	1			1
Caen, France	1			1
Calgary, United States of America	1			1
Dax, France	1			1
Grenoble, France	1			1
Essen, Germany	1	1		
Frankfurt, Germany	1		1	
Jerusalem, Israel	1			1
Lausanne, Switzerland	1			1
Le Havre, France	1			1
Lübeck, Germany	1	1		
Mannheim, Germany	1			1
New York, United States of America	1			1
Rodez/Vichy, France	1			1
Toulouse/Brives, France	1			1
Toulouse/Tarbes, France	1			1
Tours, France	1		1	
Ulm, Germany	1	1		

Overview of treatment centers. Within 39 centers, 111 patients were treated with ICI. The centers are listed based on the total number treated, followed by alphabetical order.

eTable 2: Overview of positive ELISpot findings

© 2026 Möhn N. et al. *JAMA Neurology*.

No.	Unit	Negative control	Positive control	BKV_LT Pool	BKV_VP1 Pool	JCV_LT Pool	JCV_VP1 Pool
1	Spots/5,0x10^5 PBMCs	0	TNTC	4.5	14.5	2	14.5
	Spots/10,000 CD3+	0	TNTC	0.11	0.37	0.05	0.37
2	Spots/5.0x10^5 PBMCs	7	700	27.5	9.5	7.5	50
	Spots/10,000 CD3+	0.74	73.53	2.89	1	0.79	5.25
3	Spots/5.0x10^5 PBMCs	3	TNTC	1	22.5	6.5	46.0
	Spots/10,000 CD3+	0.09	TNTC	0.03	0.7	0.2	1.42
4	Spots/5.0x10^5 PBMCs	0	TNTC	2	2	2.5	3.5
	Spots/10,000 CD3+	0	TNTC	0.05	0.05	0.06	0.09
5	Spots/5.0x10^5 PBMCs	0	TNTC	1.5	3.5	11.5	7.0
	Spots/10,000 CD3+	0	TNTC	0.04	0.09	0.29	0.18
6	Spots/5.0x10^5 PBMCs	0	539.5	1.5	1.5	4.5	6.0
	Spots/10,000 CD3+	0	14.71	0.04	0.04	0.12	0.16
7	Spots/5.0x10^5 PBMCs	1	TNTC	17	12	Pooled: 57	
	Spots/10,000 CD3+	0.04	TNTC	0.75	0.53	Pooled: 2.5	
8	Spots/5.0x10^5 PBMCs	1.5	TNTC	5.5	6.0	2.5	19.0
	Spots/10,000 CD3+	0.04	TNTC	0.15	0.16	0.07	0.51
9	Spots/5.0x10^5 PBMCs	0	TNTC	1	5.5	4	27
	Spots/10,000 CD3+	0	TNTC	0.03	0.15	0.11	0.75
10	Spots/5.0x10^5 PBMCs	0	593	4.5	3	3.5	0
	Spots/10,000 CD3+	0	21.32	0.16	0.11	0.13	0
11	Spots/5.0x10^5 PBMCs	0	TNTC	194.5	36.0	23.5	17.0
	Spots/10,000 CD3+	0	TNTC	6.66	1.23	0.8	0.58
12	Spots/5.0x10^5 PBMCs	0	527.5	1	5.5	1	0.5
	Spots/10,000 CD3+	0	13.84	0.03	0.14	0.03	0.01
13	Spots/5.0x10^5 PBMCs	1	TNTC	2	3	1	10.5
	Spots/10,000 CD3+	0.06	TNTC	0.12	0.18	0.06	0.62
14	Spots/5.0x10^5 PBMCs	0	753	16.5	25.5	8.5	28.5
	Spots/10,000 CD3+	0	22.28	0.49	0.75	0.25	0.84
15	Spots/5.0x10^5 PBMCs	2	725.0	9	29	16	31
	Spots/10,000 CD3+	0.05	17.13	0.21	0.69	0.38	0.73
16	Spots/5.0x10^5 PBMCs	0	926	1	24	1.5	25
	Spots/10,000 CD3+	0	32.53	0.04	0.84	0.05	0.88
17	Spots/10^6 PBMC	1	560.5	N/A	N/A	11	

Overview of positive ELISpot findings. No.: number; JCV: JC virus; BKV: BK virus; LT: large T (LT) protein; VP1: viral protein 1; PBMC: Peripheral Blood Mononuclear Cells; TNTC: too numerous to count; N/A: not applicable.

eTable 3: Outcome predictors of therapeutic response in the overall cohort

© 2026 Möhn N. et al. *JAMA Neurology*.

Predictor	Odds Ratio (OR)	95% CI (OR)	p-value
Age (per year)	0.9887	0.9618 to 1.016	0.4067
mRS (per 1-point increase)	0.8632	0.5967 to 1.239	0.4251
JCV viral load prior to treatment	0.999997	0.999991 to 1.00000	**0.0074**
MRI lesions with contrast enhancement pre treatment	2.044	0.8394 to 5.180	0.1159
PML-IRIS	0.9947	0.3443 to 2.831	0.992

Outcome predictors in the overall cohort. The predictor JC viral load prior to treatment was associated with a statistically significant but negligible effect on the outcome (OR 0.999997, 95% CI 0.999991–0.999999, p = 0.0074). Subsequent analysis of predictors indicated no significant influence. mRS: modified Rankin Scale; JCV: John Cunningham virus; MRI: magnet resonance imaging; PML: progressive multifocal leukoencephalopathy; IRIS: immune reconstitution inflammation syndrome.

eTable 4: Outcome predictors of therapeutic response within the unknown T-cell cohort

Predictor	Odds Ratio (OR)	95% CI (OR)	p-value
Age (per year)	0.9828	0.9477 to 1.018	0.3286
mRS (per 1-point increase)	0.5885	0.3485 to 0.9461	**0.0281**
JC viral load pre treatment	0.999998	0.999993 to 1.00000	0.1362
MRI lesion with contrast enhancement pre treatment	1.534	0.355 to 3.69	0.809
PML-IRIS	0.755	0.205 to 2.57	0.6556

Outcome predictors within the unknown T-cell cohort. Higher baseline disability, as measured by the modified Rankin Scale (mRS), was associated with lower odds of treatment response (OR per 1-point increase 0.589, 95% CI 0.349–0.946, p = 0.028). Subsequent analysis of predictors indicated no significant influence. JCV: John Cunningham virus; MRI: magnet resonance imaging; PML: progressive multifocal leukoencephalopathy; IRIS: immune reconstitution inflammation syndrome.

eTable 5: Adverse events

Adverse events	JCV/BKV-specific T cells positive (n=21)	JCV/BKV-specific T cells negative (n=22)	JCV/BKV-specific T-cell status unknown (n=68)
Psoriasis, no. (%)	2 (10)	0 (0)	2 (3)
Rash (not psoriatic), no. (%)	0 (0)	2 (9.5)	2 (3)
Fever/infectious symptoms, no. (%)	0 (0)	3 (14)	4 (6)
Gastrointestinal symptoms, no. (%)	0 (0)	2 (9.5)	6 (9)
Haematological symptoms, no. (%)	0 (0)	1 (5)	2 (3)
Cardiovascular symptoms, no. (%)	0 (0)	1 (5)	2 (3)
Others*, no. (%)	0 (0)	1 (5)	5 (7)
Not assessed, (%)	1 (5)	2 (9)	0 (0)

© 2026 Möhn N. et al. *JAMA Neurology*.

Overview of adverse events under ICI therapy. In the group of patients with unknown T cell status, n=3 patients had more than one adverse event. *Others: oligoarthritis (n=1), vitiligo (n=1), anaphylaxis (n=1), pneumonitis (n=1), myositis (n=1), leg edema (n=1). Abbreviations: JCV: JC virus; BKV: BK virus.

eREFERENCES

1. Bonifacius A, Lamottke B, Tischer-Zimmermann S, et al. Patient-tailored adoptive immunotherapy with EBV-specific T cells from related and unrelated donors. J Clin Invest. 2023;133(12):e163548. doi: 10.1172/JCI163548.
2. Sukdolak C, Tischer S, Dieks D, et al. CMV-, EBV- and ADV-specific T cell immunity: screening and monitoring of potential third-party donors to improve post-transplantation outcome. Biol Blood Marrow Transplant. 2013;19(10):1480–1492. doi: 10.1016/j.bbmt.2013.07.015.
3. Tischer S, Priesner C, Heuft H, et al. Rapid generation of clinical-grade antiviral T cells: selection of suitable T-cell donors and GMP-compliant manufacturing of antiviral T cells. J Transl Med. 2014;12:336. doi: 10.1186/s12967-014-0336-5.
4. Schulze Lammers FC, Bonifacius A, Tischer-Zimmermann S, et al. Antiviral T-Cell Frequencies in a Healthy Population: Reference Values for Evaluating Antiviral Immune Cell Profiles in Immunocompromised Patients. J Clin Immunol. 2022;42(3):546–558. doi: 10.1007/s10875-021-01205-1

© 2026 Möhn N. et al. *JAMA Neurology*.
