# Supplementary material for: Virus-Specific T Cells and Response to Checkpoint Inhibitors in Progressive Multifocal Leukoencephalopathy
Source: JAMA Neurol. 2026 Jan 20;83(3):280–9. doi: 10.1001/jamaneurol.2025.5318 (PMC12820779; doi:10.1001/jamaneurol.2025.5318)
Supplement: Supplement 2. — Immunotherapy for PML Study Group members [file jamaneurol-e255318-s002.pdf]

Supplemental Online Content: Nonauthor Collaborators
*First name, last name, and suffix (if applicable) are required and will appear in PubMed.

*Group Name(s): Immunotherapy for PML study group							
***First Name and Middle Initial(s)**	***Last Name**	***Suffix (eg, Jr, III)**	Academic Degrees	Institution	Location (city, state/province, country)	Role or Contribution, eg, chair, principal investigator	Group (if more than 1 Group listed in the byline) and/or Subgroup (eg, Steering Committee)
Laurence	De Menibus		MD	Le Havre Hospital	Le Havre, France	Investigator	IT PML study group
Damien	Roos-Weil		MD	APHP	Paris, France	Investigator	IT PML study group
Juliette Rakotoaris	Rakotoarison		MD	Tours University Hospital	Tours, France	Investigator	IT PML study group
Yann	Leveneur		MD	Tarbes Hospital	Tarbes, France	Investigator	IT PML study group
Niklas	Grassl		MD	DKTK CCU Neuroimmunology and Bra	Heidelberg, Germany	Investigator	IT PML study group
Francois	Lifermann		MD	Dax Hospital	Dax, France	Investigator	IT PML study group
Fleur	Cohen-Aubart		MD	APHP	Paris, France	Investigator	IT PML study group
Douglas	Ney		MD	UCHealth Cancer Care - Anschutz Me	Aurora, USA	Investigator	IT PML study group
Ronak	Kapadia		MD	Cumming School of Medicine	Calgary, Canada	Investigator	IT PML study group
Yasemin	Goreci		MD	University Hospital of Cologne	Cologne, Germany	Investigator	IT PML study group
Yael	Dinur-Schejter		MD	University of Alberta	Alberta, Canada	Investigator	IT PML study group
Tzlil	Shifman		MD	Hadassah Medical Center	Jerusalem, Izrael	Investigator	IT PML study group
Oded	Shamriz		MD	Hadassah Medical Center	Jerusalem, Izrael	Investigator	IT PML study group
Joseph	Berger		MD	University of Pennsylvania	Pennslyvania, USA	Investigator	IT PML study group
Olivier	Lambotte		MD	APHP	Paris, France	Investigator	IT PML study group
Asaff	Harel		MD	Zucker School of Medicine at Hofstra	Uniondale, NY, USA	Investigator	IT PML study group
Benjamin	Wyplosz		MD	APHP	Paris, France	Investigator	IT PML study group
Bodo	Grimbacher		MD	Freiburg University Hospital	Freiburg, Germany	Investigator	IT PML study group
Martijn	Wijburg		MD	Amsterdam UMC	Amsterdan, Holland	Investigator	IT PML study group
Matthijs	Brouwer		MD	Amsterdam UMC	Amsterdan, Holland	Investigator	IT PML study group
Xavier	Engalenc		MD	Brives Hospital	Brives, France	Investigator	IT PML study group
Marion	Gaudin		MD	Brives Hospital	Brives, France	Investigator	IT PML study group
Clemens	Kupper		MD	Munich University Hospital	Munich, Germany	Investigator	IT PML study group
Achille	Aouba		MD	Caen University Hospital	Caen, France	Investigator	IT PML study group
Victoria	Manda		MD	APHP	Paris, France	Investigator	IT PML study group
Xavier	Brousse		MD	Bordeaux University Hospital	Bordeaux, France	Investigator	IT PML study group
Mailys	Ducours		MD	Bordeaux University Hospital	Bordeaux, France	Investigator	IT PML study group

Supplemental Online Content: Nonauthor Collaborators
*First name, last name, and suffix (if applicable) are required and will appear in PubMed.

***First Name and Middle Initial(s)**	***Last Name**	***Suffix (eg, Jr, III)**	Academic Degrees	Institution	Location (city, state/province, country)	Role or Contribution, eg, chair, principal investigator	Group (if more than 1 Group listed in the byline) and/or Subgroup (eg, Steering Committee)
Pierre	Duffau		MD	Bordeaux University Hospital	Bordeaux, France	Investigator	IT PML study group
Jean Christophe	Ouallet		MD	Bordeaux University Hospital	Bordeaux, France	Investigator	IT PML study group
Hela	Mrabet		MD	Rodez Hospital	Rodez, France	Investigator	IT PML study group
Florence	Gourdon		MD	Vichy Hospital	Vichy, France	Investigator	IT PML study group
Marion	Le Maréchal		MD	Grenoble University Hospital	Grenoble, France	Investigator	IT PML study group
Raphael	Bernard-Valnet		MD	Lausanne University Hospital	Lausanne, Switzerland	Investigator	IT PML study group
Pierre	Delobel		MD	Toulouse University Hospital	Toulouse, France	Investigator	IT PML study group
Rebecca	Lajaunie		MD	Toulouse University Hospital	Toulouse, France	Investigator	IT PML study group
Nassim	Kamar		MD	Toulouse University Hospital	Toulouse, France	Investigator	IT PML study group
Marine	Joly		MD	Toulouse University Hospital	Toulouse, France	Investigator	IT PML study group
Emmanuel	Treiner		MD	Toulouse University Hospital	Toulouse, France	Investigator	IT PML study group
Sebastien	Lhomme		MD	Toulouse University Hospital	Toulouse, France	Investigator	IT PML study group
Fabrice	Bonneville		MD	Toulouse University Hospital	Toulouse, France	Investigator	IT PML study group
Carole	Ribaute		MD	Toulouse University Hospital	Toulouse, France	Investigator	IT PML study group
Jonathan	Ciron		MD	Toulouse University Hospital	Toulouse, France	Investigator	IT PML study group
Damien	Biotti		MD	Toulouse University Hospital	Toulouse, France	Investigator	IT PML study group
Xavier	Boumaza		MD	Toulouse University Hospital	Toulouse, France	Investigator	IT PML study group
Baptiste	Bonneau		MSc	Toulouse University Hospital	Toulouse, France	Investigator	IT PML study group
Agnès	Sommet		MD	Toulouse University Hospital	Toulouse, France	Investigator	IT PML study group
Béatrice	Pignolet		PhD	Toulouse University Hospital	Toulouse, France	Investigator	IT PML study group
