# Supplementary material for: Virus-Specific T Cells and Response to Checkpoint Inhibitors in Progressive Multifocal Leukoencephalopathy
Source: JAMA Neurol. 2026 Jan 20;83(3):280–9. doi: 10.1001/jamaneurol.2025.5318 (PMC12820779; doi:10.1001/jamaneurol.2025.5318)
Supplement: Supplement 3. — Data sharing statement [file jamaneurol-e255318-s003.pdf]

Data Sharing Statement

Data
Data available: No

Additional Information
Explanation for why data not available: To protect the privacy and rights of the individual participants, the data supporting the findings of this study are available only upon reasonable request. Data will be shared in an anonymized format and in compliance with applicable data protection regulations. This controlled access approach ensures that sensitive personal health information is safeguarded and not inadvertently disclosed.
